# Clinicians’ Tendencies to Under-Rate Parkinsonian Tremors in the Less Affected Hand

**DOI:** 10.1371/journal.pone.0131703

**Published:** 2015-06-25

**Authors:** Hong Ji Lee, Sang Kyong Kim, Hyeyoung Park, Han Byul Kim, Hyo Seon Jeon, Yu Jin Jung, Eungseok Oh, Hee Jin Kim, Ji Young Yun, Beom S. Jeon, Kwang Suk Park

**Affiliations:** 1 The Interdisciplinary Program for Bioengineering, Seoul National University, Seoul, Republic of Korea; 2 The Department of Neurology and Movement Disorder Center, Seoul National University Hospital, Seoul, Republic of Korea; 3 The Department of Neurology, Kyung Hee University Hospital at Gangdong, Seoul, Republic of Korea; 4 The Department of Neurology, Chungnam National University Hospital, Daejeon, Republic of Korea; 5 The Department of Neurology, Konkuk University Hospital, Seoul, Republic of Korea; 6 The Department of Neurology, Ewha Womans University Mokdong Hospital, Seoul, Republic of Korea; 7 The Department of Biomedical Engineering, Seoul National University, Seoul, Republic of Korea; National Institute of Health, UNITED STATES

## Abstract

The standard assessment method for tremor severity in Parkinson’s disease is visual observation by neurologists using clinical rating scales. This is, therefore, a subjective rating that is dependent on clinical expertise. The objective of this study was to report clinicians’ tendencies to under-rate Parkinsonian tremors in the less affected hand. This was observed through objective tremor measurement with accelerometers. Tremor amplitudes were measured objectively using tri-axis-accelerometers for both hands simultaneously in 53 patients with Parkinson’s disease during resting and postural tremors. The videotaped tremor was rated by neurologists using clinical rating scales. The tremor measured by accelerometer was compared with clinical ratings. Neurologists tended to under-rate the less affected hand in resting tremor when the contralateral hand had severe tremor in Session I. The participating neurologists corrected this tendency in Session II after being informed of it. The under-rating tendency was then repeated by other uninformed neurologists in Session III. Kappa statistics showed high inter-rater agreements and high agreements between estimated scores derived from the accelerometer signals and the mean Clinical Tremor Rating Scale evaluated in every session. Therefore, clinicians need to be aware of this under-rating tendency in visual inspection of the less affected hand in order to make accurate tremor severity assessments.

## Introduction

Tremor is defined as the involuntary and rhythmic oscillatory movement of a body part [1]. Generally, tremor severities are subjectively assessed by clinicians through visual observation using clinical rating scales during neurological examination [2]. Various clinical rating scales are used to quantify resting, postural, and action tremors such as the Fahn-Tolosa-Marin Tremor Rating Scale (FTM TRS), the Unified Parkinson’s Disease Rating Scale (UPDRS), and the Clinical Tremor Rating Scale (CTRS) [3–6]. They provide clinicians with systems to rate the tremor severity of a body part during rest and movement on a five-point scale. However, since these are discrete and subjective ratings influenced by clinical expertise and personal bias [4], there may be inter- and intra-individual differences.

Several researchers developed tremor assessment methods using a variety of sensing technologies such as accelerometers, actigraphs, gyroscopes, electromagnetic tracking, and electromyography [2, 7–19]. These objective tests showed high correlations with clinical rating scales and high accuracy with low errors using analytical classifiers [8, 9, 12]. Moreover, a system integrating an accelerometer and a gyroscope has been used as a commercial product [9].

This paper aimed to demonstrate neurologists’ tendencies to under-rate Parkinsonian tremors in the less affected hand when the contralateral hand had severe tremor. In addition, we were particularly interested in whether this tendency was due to less attention to the less affected side, and whether the tendency was frequent among neurologists. Therefore, tremor in patients with Parkinson’s disease (PD) was measured by compact tri-axis-accelerometers from both hands simultaneously. The tremor measured by the accelerometer was compared with clinical rating scales rated by neurologists.

## Methods

### Patients

The study was approved by the Institutional Review Board of Seoul National University Hospital (IRB No. D-1202-078-398). Patients with tremor dominant PD who visited the Movement Disorder Clinic at Seoul National University Hospital participated in this study (November 2012 to February 2013). Patients with leg tremors or dyskinesias were excluded. Fifty-three patients (28 males, 25 females, 66.6 ± 8.3 years old) were enrolled. All patients with PD met the diagnostic criteria of the United Kingdom Parkinson’s Disease Society Brain Bank guidelines. All patients provided written informed consent prior to study participation. Even when patients had unilateral tremor symptoms, data were acquired from both hands.

### Experimental Protocol

Tri-axis-accelerometer sensors (LIS3LV02DQ, STMicroelectronics N.V. Switzerland) that can measure up to ± 6 g on the X, Y, and Z axes were used. The sampling rate was 129 Hz and the accelerometer signals were transmitted to a laptop via Bluetooth. After patients were seated on a chair with a backrest, the tri-axis-accelerometer sensors were attached over the distal phalanges of the middle finger of each hand. If the tremor symptom was severe on the thumb or index finger, the sensor position was changed to the more severe finger. Each patient performed resting and postural tremor tests. A video camera (Panasonic HDC-TM700, 1920 × 1080 p HD at 60 frames per second) was also installed in front of the patient to record the tasks simultaneously. The video closely recorded both hands. Resting tremor was recorded for 30 seconds while the patient sat comfortably at rest with both hands on their lap. Postural tremor was also measured for 30 seconds with both arms stretched forward against gravity.

Four neurologists from four hospitals participated in visual rating sessions of the tremor severity of right and left hands separately with the CTRS while watching the videotaped resting and postural tremors for 30 seconds. All of the neurologists had at least two years of subspecialty training in movement disorders.

The CTRS was used to assess the tremor severity of a body part using scores of 0 to 4, where 0 = none; 1 = intermittent and barely perceivable; 2 = intermittent and amplitude < 2 cm; 3 = continuous and amplitude 2 to 4 cm; 4 = severe and amplitude > 4 cm [4]. Only integer rating scores were allowed during the rating.

#### Session I

Two neurologists rated the videotaped tremors with the CTRS.

#### Session II

Six months after Session I, we informed the participating neurologists of this under-rating tendency and asked them to re-rate the same videotapes. The videotapes were randomly rearranged. The raters were blind to the previous scores.

#### Session III

To examine whether this under-rating tendency is frequent among neurologists, two other neurologists who were not informed of this tendency were requested to rate the videotaped tremors with the same rating scales.

### Signal Processing and Statistical Analysis

Outliers (more than four standard deviations [SD] from the mean) were removed from the accelerometer signals to eliminate abrupt artifact motion interference. Then, each axis signal was filtered with a range of 1–20 Hz. After being scaled to cm/s^2^, the signals were integrated twice. The vector, d(i) = [d_x_(i), d_y_(i), d_z_(i)], with i denoting discrete time, was formed to calculate displacements. The root mean square (RMS) of displacement was calculated for time-based measurement. This method was applied to both resting and postural tremors.

Linear regression models were used to estimate scores from the RMS amplitudes. The estimated scores were compared with the mean CTRS scores using Cohen’s kappa for each task. The agreements between the ratings were calculated by the weighted kappa on nine-point scales from zero to four in 0.5 intervals. Cohen’s kappa is a statistical measure of rater agreement for nominal scales. The kappa differs from other agreement measures in that it considers the agreement expected by chance. Especially for ordered categories, kappa is weighted to account for the degree of disagreement by emphasizing large differences between ratings [20–21].

For statistical analysis, the data were divided into two groups. Group 1 contained the mean RMS amplitudes of the hands rated CTRS 0 when the severity of the contralateral hand was under mean CTRS 1.5. Group 2 was the mean RMS amplitudes of the hands rated CTRS 0 when the severity of the contralateral hand was over mean CTRS 2. The mean RMS amplitudes between Groups 1 and 2 were compared with two-tailed independent samples t-tests.

All offline analyses were done by MATLAB R2013b (MATLAB, Mathworks, USA).

## Results

### Session I

**Fig 1:**
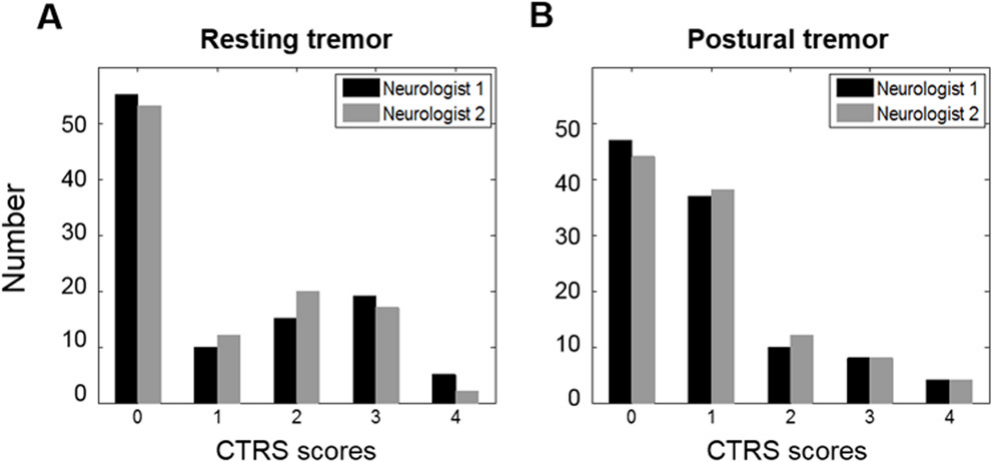
Distribution of data in Session I. The number of the CTRS scores of two neurologists for (A) resting and (B) postural tremor tasks.

The distribution of CTRS scores for each tremor task in Session I is shown in Fig 1. Because the symptoms of each hand were evaluated separately by patient, there were a total of 106 tremor severity measurements with a score range of 0–4. The inter-rater agreements were 0.96 for resting tremor and 0.93 for postural tremor. The kappa agreements for nine severity ratings between the estimated scores under the linear regression model and the mean CTRS scores of the two neurologists were 0.90 for resting tremor and 0.83 for postural tremor in Session I.

**Fig 2:**
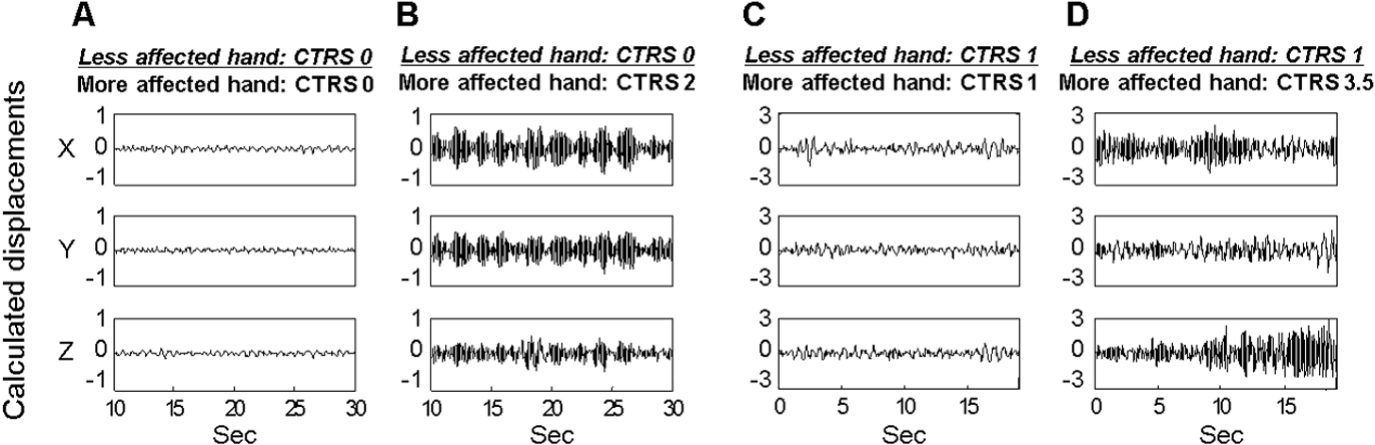
Tremor signals by accelerometer in the less affected hand across various CTRS scores of the contralateral hand. Calculated displacement signals in each axis measured from: (A) the less affected hand when both hands were rated CTRS 0; (B) the less affected hand rated CTRS 0 when the more affected hand was rated CTRS 2; (C) the less affected hand when both hands were rated CTRS 1; (D) the less affected hand rated CTRS 1 when the more affected hand was rated CTRS 3.5. The amplitudes of displacements in the hands rated with the same CTRS differed depending on the contralateral hand’s severity.

However, the neurologists’ tendencies to under-rate the less affected hand were noted when the contralateral hand had severe tremor. Displacement signals in each axis calculated from the accelerometer signals are shown in Fig 2, across mean CTRS scores rated by the two neurologists. Although the less affected hands of two patients were rated CTRS 0, the displacement signals in Fig 2B were considerably larger in all axes than those in Fig 2A. Similarly, even though the less affected hands of two patients were rated CTRS 1, the displacements in Fig 2D were larger in all axes than those in Fig 2C. In other words, the tremor severity of the less affected hand was under-rated when the contralateral hand had severe tremor (Fig 2B and 2D). The same tendency was found in 75.9% (22/29 patients) of data sets where the mean CTRS difference between both hands was over 2. Moreover, those findings from right and left hand were 36.4% and 63.6%, respectively.

**Fig 3:**
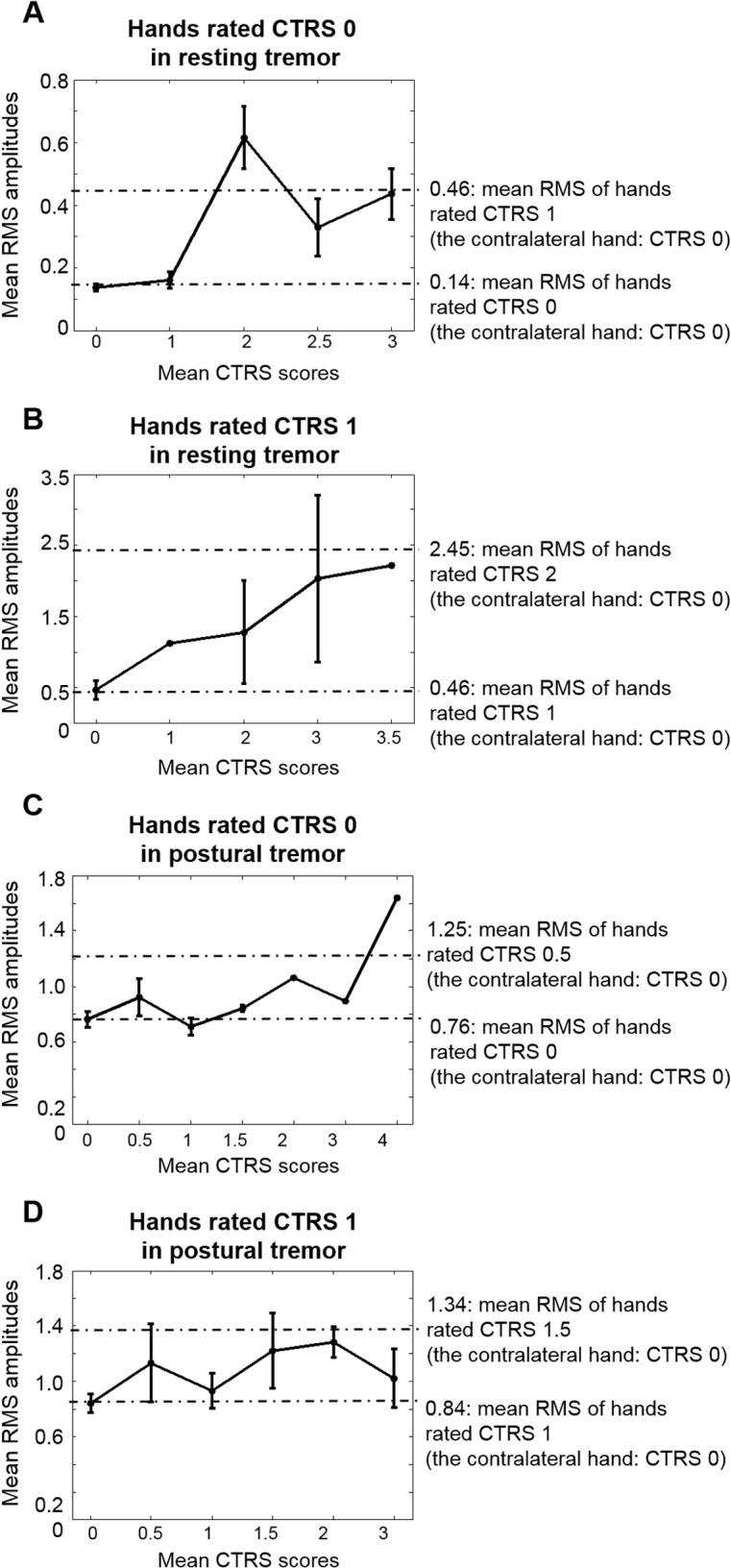
Mean RMS amplitudes in Session I. The mean RMS amplitudes and standard errors of the hands rated with the same CTRS score, across the severity of the contralateral hand. (A) The mean RMS amplitudes of the hands rated CTRS 0 in resting tremor were 0.14 ± 0.04, 0.16 ± 0.05, 0.62 ± 0.34, 0.33 ± 0.20, and 0.44 ± 0.21 when the contralateral hand’s severity ratings were 0, 1, 2, 2.5, and 3, respectively. (B) The mean RMS amplitudes of the hands rated CTRS 1 in resting tremor were 0.46 ± 0.27, 1.12 ± 0.00, 1.28 ± 1.02, 2.03 ± 1.66, and 2.21 ± 0.00 when the contralateral hand’s severity ratings were 0, 1, 2, 3, and 3.5, respectively. (C) The mean RMS amplitudes of the hands rated CTRS 0 in postural tremor were 0.76 ± 0.18, 0.92 ± 0.30, 0.71 ± 0.20, 0.84 ± 0.03, 1.06 ± 0.00, 0.89 ± 0.00 and 1.64 ± 0.00 when the contralateral hand’s severity ratings were 0, 0.5, 1, 1.5, 2, 3, and 4, respectively. (D) The mean RMS amplitudes of the hands rated CTRS 1 in postural tremor were 0.84 ± 0.21, 1.13 ± 0.63, 0.93 ± 0.18, 1.22 ± 0.47, 1.28 ± 0.22, and 1.02 ± 0.37 when the contralateral hand’s severity ratings were 0, 0.5, 1, 1.5, 2, and 3, respectively.

To examine this tendency systematically, the distributions of the mean RMS amplitudes of the hands rated CTRS 0 or CTRS 1 are shown in Fig 3, by the mean CTRS scores of contralateral hand. In Fig 3A, when the contralateral hand had severe tremor, the mean RMS amplitudes of CTRS 0, 0.62, 0.33, and 0.44, were in the range of those of mean CTRS 0.5–1. In Fig 3B, when the contralateral hand had severe tremor, the mean RMS amplitudes of CTRS 1, 1.28, 2.03, and 2.21, were in the range of those of mean CTRS 1.5–2. In other words, the severity of the less affected hand was rated lower in resting tremor when the severity of the contralateral hand was severe. For resting tremor, there was significant difference in the mean RMS amplitudes between Group 1 and Group 2, respectively (2-tailed independent samples t-test, p < 0.05).

However, as shown in Fig 3C and 3D, this tendency was not seen in postural tremors except for a patient who had CTRS 0 on one hand and CTRS 4 on the other. For postural tremor, there was no significant difference in the mean RMS values between Groups 1 and 2, respectively (2-tailed independent samples t-test, p > 0.05).

### Session II

The inter-rater agreements were 0.85 for resting tremor and 0.80 for postural tremor in Session II. The kappa agreements for nine severity ratings between the estimated scores from RMS amplitudes and mean CTRS scores of the two neurologists were 0.93 for resting tremor and 0.85 for postural tremor in Session II.

**Fig 4:**
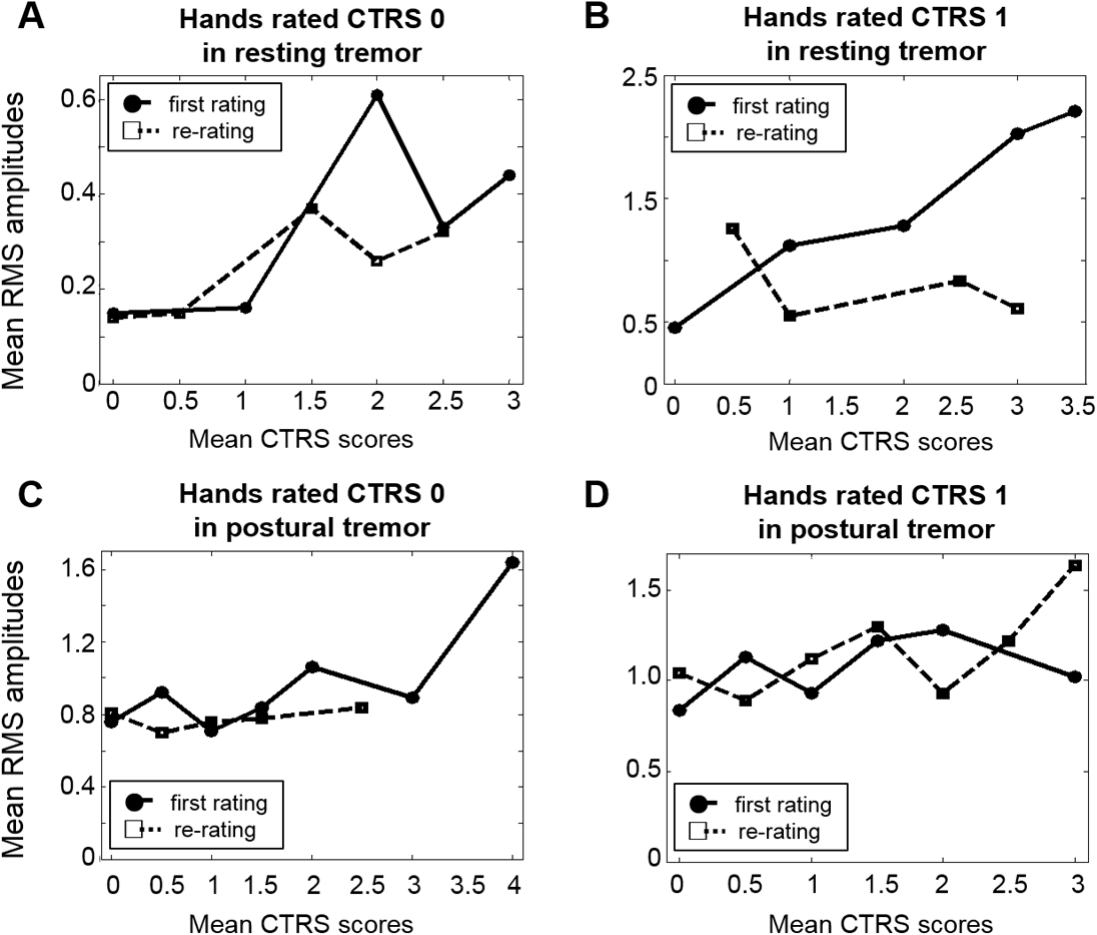
Mean RMS amplitudes in Session I and II. The distributions of mean RMS amplitudes of the hands rated at the same CTRS, by the severity of the contralateral hand, for each tremor in Session I (circle and solid line) and Session II (square and dashed line). The RMS amplitudes of the hands rated: (A) CTRS 0 and (B) CTRS 1 in resting tremor; (C) CTRS 0 and (D) CTRS 1 in postural tremor.

In Fig 4, the results of the re-ratings were compared with the ratings in Session I. The mean RMS amplitudes of the less affected hand were considerably lower in Session II than in Session I in resting tremor, when the contralateral hand had severe tremor (Fig 4A and 4B). In summary, the RMS amplitudes of the less affected hand rated at the same CTRS were similar regardless of the severity of the more affected hand in resting tremor in Session II. On the other hand, there were no differences between Session I and Session II in postural tremors (Fig 4C and 4D).

### Session III

The inter-rater agreements in Session III were 0.89 for resting tremor and 0.84 for postural tremor. Moreover, the kappa agreements between the first and third raters were 0.92 for resting tremor and 0.81 for postural tremor. The agreements for nine severity ratings between the estimated scores and mean CTRS scores of the two neurologists were 0.94 for resting tremor and 0.84 for postural tremor in Session III.

**Fig 5:**
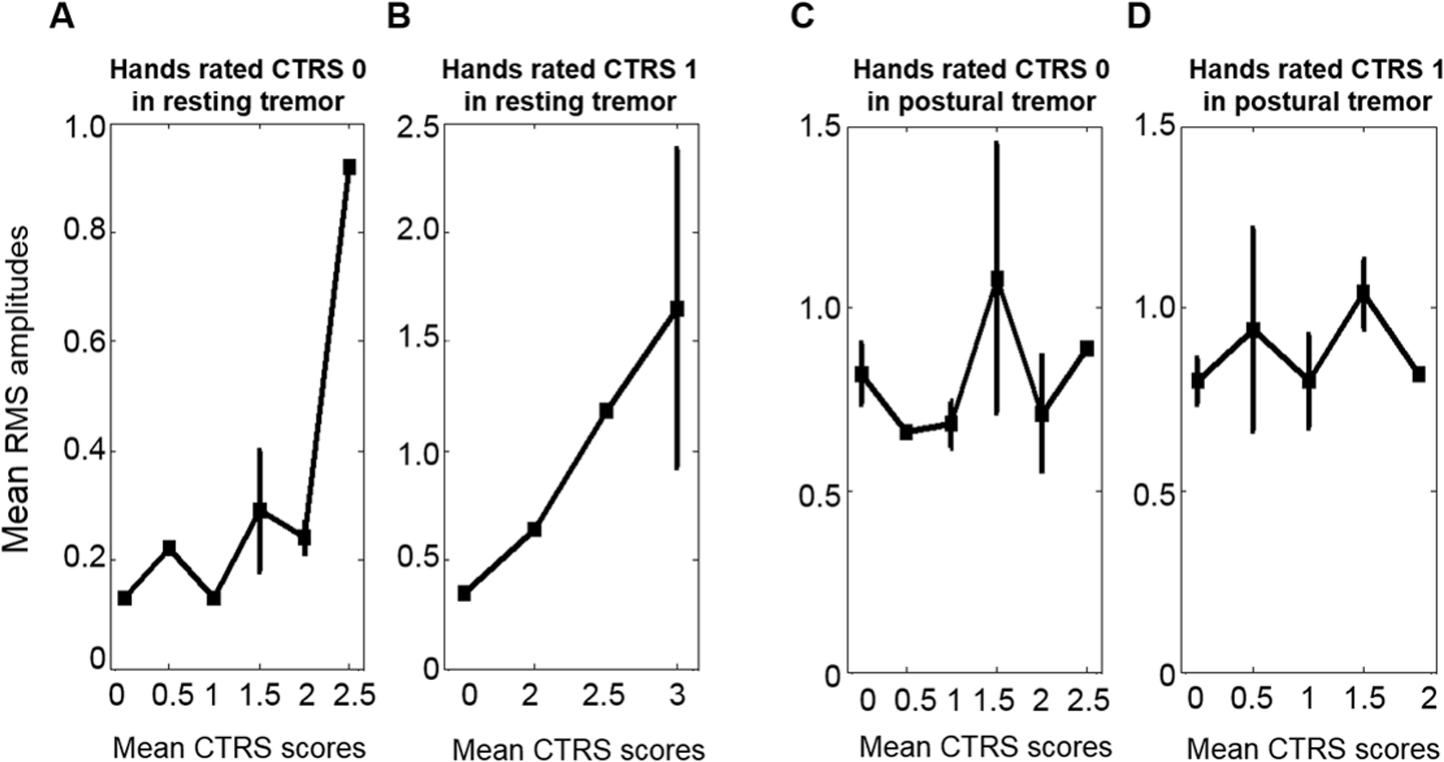
Mean RMS amplitudes in Session III. The mean RMS amplitudes and standard errors of the hands rated with the same CTRS score, by the severity of the contralateral hand. (A) The mean RMS values of the hands rated CTRS 0 in resting tremor were 0.13 ± 0.03, 0.22 ± 0.00, 0.13 ± 0.00, 0.29 ± 0.19, 0.24 ± 0.10, and 0.92 ± 0.00 when the mean CTRS scores of the contralateral hand were 0, 0.5, 1, 1.5, 2, and 2.5, respectively. (B) The mean RMS amplitudes of the hands rated CTRS 1 in resting tremor were 0.35 ± 0.00, 0.64 ± 0.00, 1.18 ± 0.00, and 1.65 ± 1.26 when the mean CTRS scores of the contralateral hand were 0, 2, 2.5, and 3, respectively. (C) The mean RMS amplitudes of the hands rated CTRS 0 in postural tremor were 0.82 ± 0.24, 0.66 ± 0.00, 0.68 ± 0.16, 1.08 ± 0.52, 0.71 ± 0.22, and 0.89 ± 0.00 when the mean CTRS scores of the contralateral hand were 0, 0.5, 1, 1.5, 2, and 2.5, respectively. (D) The mean RMS amplitudes of the hands rated CTRS 1 in postural tremor were 0.80 ± 0.15, 0.94 ± 0.55, 0.80 ± 0.18, 1.04 ± 0.23, and 0.82 ± 0.00 when the mean CTRS scores of the contralateral hand were 0, 0.5, 1, 1.5, and 2, respectively.

The mean RMS amplitudes of Session III are in Fig 5 and show essentially the same tendency as those from Session I.

## Discussion

In this study, tremors of patients with PD were measured using tri-axis-accelerometer sensors from both hands simultaneously. The videotaped tremors were rated by neurologists across three rating sessions. The interesting finding was that neurologists tended to under-rate rest tremor in the less affected hand when the contralateral hand had severe tremor in Session I. In other words, neurologists rated the tremor severity of the less affected hand lower when the contralateral hand had severe tremor. We assumed that this tendency was due to less attention to the less affected side because it was corrected during a re-rating that occurred after the raters were informed of the under-rating tendency in Session II. Moreover, since this under-rating tendency was repeated by other neurologists in Session III, it appeared that this tendency was general.

The ratings were very consistent because the inter-rater agreements were high. The inter-rater agreement statistics (kappa) were above 0.80, indicating almost perfect agreement in Sessions I, II, and III. Moreover, the agreements between the estimated scores derived from the accelerometer data and mean CTRS scores by two neurologists were above 0.82 for each tremor in all rating sessions. These results are quite similar to performances reported by other studies [4, 8, 9, 12].

The rating of tremor severity based on the videotapes could be influenced by the video protocols and rating environment such as camera angle, image size, the distance between observers and the video screen, and video players [11]. We used video camera with full HD at 60 frames per second to reduce loss of information on fast tremor frequency. Moreover, the video in this study focused on both hands to rate the severities more precisely.

The tendency to under-rate the less affected hand was found only for resting tremor, not for postural tremor. Since the consistency of inter-rater reliability for both tremor tasks was guaranteed, potential differences in video recording quality and readings between resting and postural tremor did not explain why this tendency was seen only for resting tremor. As shown in Figs 3, 4 and 5, the RMS amplitudes of the hands rated CTRS 0 for postural tremor were larger than those for resting tremor. When the accelerometer signals of CTRS 0 in postural tremor were filtered with a high-pass filter of 2.5 Hz, the mean RMS amplitude was 0.26. It was markedly reduced from 0.76 compared to when a 1 Hz high-pass filter was used (Fig 3C and 3D). When the accelerometer signals were filtered with a low-pass filter of around 8 Hz, the mean RMS values did not differ significantly from when no low-pass filter was applied, supporting the fact that high frequency components did not contribute to the calculated RMS amplitudes. Therefore, it appears to be due to low frequency-high amplitude movement components such as perturbation and drift while stretching arms forward against gravity for 30 s. Part of this background noise may be included in spite of filtering at 1 Hz during the calculation for postural tremor.

Since patients’ hands were on their laps during resting tremor task, patients with leg tremors were excluded. In addition, outlier removal and filtering methods were used to eliminate the effect of abrupt motion artifact or body sway. Despite patient selection and signal processing, there was a limitation that the accelerometer could capture tremors that may not be easily perceived by the clinicians, because the sensitivity of accelerometer was different from that of clinician’s eyes in rating tremor.

Bain et al. used a sample size of 20 patients and four raters for validation of a clinical rating scale [11]. Stacy et al. evaluated inter-rater and intra-rater reliability of FTM TRS with 17 patients with essential tremor and 59 raters [4]. Goetz et al. recruited 877 patients with PD from 39 sites and 69 raters from 39 treatment centers to assess the Movement Disorder Society UPDRS (MDS-UPDRS) [6]. When compared to other studies, the sample size of four neurologists from four hospitals who contributed for the rating of the videotapes in this study was quite small. Therefore, generalization of these findings is limited.

In conclusion, clinicians who use clinical scales in their daily routine may need to be aware of this under-rating tendency in the less affected hand when the contralateral hand had severe tremor. Moreover, accelerometer measurements may help clinicians rate tremor severity during clinical trials to make clinician’s assessments more consistent and reproducible across different raters and centers. To be used in clinical practice, however, it requires future studies because the use of accelerometers as the standardized guideline in the clinical management for tremor of PD patients is still questionable.

## References

[1] Findley LJ, Koller WC. Definitions and Behavioural Classification. In: Findley LJ, Koller WC, editors. Handbook of Tremor Disorders; 1995. pp. 1–5.

[2] Smeja M, Foerster F, Fuchs G, Emmans D, Hornig A, Fahrenberg J. 24-h assessment of tremor activity and posture in Parkinson’s disease by multi-channel accelerometry. J Psychophysiol. 1999;13: 245–256.

[3] Fahn S, Tolosa E, Marin C. Clinical Rating Scale for Tremor, 2nd ed. In: Jankovic J, Tolosa E, Eds. Parkinson’s disease and Movement Disorders; 1993. pp. 271–280.

[4] Stacy MA, Elble RJ, Ondo WG, Wu SC, Hulihan J, TRS Study Group. Assessment of interrater and intrarater reliability of the Fahn-Tolosa-Marin Tremor Rating Scale in essential tremor. Mov Disord. 2007;22: 833–838. 17343274 10.1002/mds.21412

[5] Movement Disorder Society Task Force on Rating Scales for Parkinson’s Disease. The Unified Parkinson’s disease rating scale (UPDRS): status and recommendations. Mov Disord. 2003;18: 738–750. 12815652 10.1002/mds.10473

[6] Goetz CG, Fahn S, Martinez-Martin P, Poewe W, Sampaio C, Stebbins GT, et al Movement Disorder Society-sponsored revision of the Unified Parkinson’s Disease Rating Scale (MDS-UPDRS): Process, format, and clinimetric testing plan. Mov Disord. 2007;22: 41–47. 17115387 10.1002/mds.21198

[7] Van Hilten JJ, Hoogland G, van der Velde EA, van Dijk JG, Kerkhof GA, Roos RA. Quantitative assessment of Parkinsonian patients by continuous wrist activity monitoring. Clin Neuropharmacol. 1993;16: 36–45. 8422656 10.1097/00002826-199302000-00004

[8] Patel S, Lorincz K, Hughes R, Huggins N, Growdon J, Standaert D, et al Monitoring motor fluctuations in patients with Parkinson’s disease using wearable sensors. IEEE Trans Inf Technol Biomed. 2009;13: 864–873. 10.1109/TITB.2009.2033471 19846382 PMC5432434

[9] Giuffrida JP, Riley DE, Maddux BN, Heldman DA. Clinical deployable Kinesia^TM^ technology for automated tremor assessment. Mov Disord. 2009;24: 723–730. 10.1002/mds.22445 19133661

[10] Calzetti S, Baratti M, Grestingy M, Findley L. Frequency/amplitude characteristics of postural tremor of the hands in a population of patients with bilateral essential tremor: implications for the classification and mechanism of essential tremor. J Neurol Neurosurg Psychiatry. 1987;50: 561–567. 3585381 10.1136/jnnp.50.5.561 PMC1031967

[11] Bain PG, Findley LJ, Atchison P, Behari M, Vidailhet M, Gresty M, et al Assessing tremor severity. J Neurol Neurosurg Psychiatry. 1993;56: 868–873. 8350102 10.1136/jnnp.56.8.868 PMC1015140

[12] Rigas G, Tzallas AT, Tsipouras MG, Bougia P, Tripoliti EE, Baga D, et al Assessment of tremor activity in the Parkinson’s disease using a set of wearable sensors. IEEE Trans Inf Technol Biomed. 2012;16: 478–487. 10.1109/TITB.2011.2182616 22231198

[13] Bacher M, Scholz E, Diener HC. 24 hour continuous tremor quantification based on EMG recording. Electroencephalogr Clin Neurophysiol. 1989;72: 176–183. 2464489 10.1016/0013-4694(89)90179-x

[14] Salarian A, Russmann H, Wider C, Burkhard PR, Vingerhoets FJ, Aminian K. Quantification of tremor and bradykinesia in Parkinson’s disease using a novel ambulatory monitoring system. IEEE Trans Biomed Eng. 2007;54: 313–322. 17278588 10.1109/TBME.2006.886670

[15] Hoff JI, Wagemans EA, van Hiltten BJ. Ambulatory objective assessment of tremor in Parkinson’s disease. Clin Neurophamacol. 2001;24: 280–283. 10.1097/00002826-200109000-00004 11586112

[16] van Someren EJ, Vonk BF, Thijssen WA, Speelman JD, Schuurman PR, Mirmiran M, et al A new actigraphy for long-term registration of the duration and intensity of tremor and movement. IEEE Trans Biomed Eng. 1998;45: 386–395. 9509754 10.1109/10.661163

[17] O’Suilleabhain PE, Dewey RB. Validation for tremor quantification of an electromagnetic tracking device. Mov Disord. 2001;16: 265–271. 11295779 10.1002/mds.1064

[18] Rajaraman V, Jack D, Adamovich SV, Hening W, Sage J, Poizner H. A novel quantitative method for 3D measurement of Parkinsonian tremor. Clin Neurophysiol. 2000;111: 338–343. 10680570 10.1016/s1388-2457(99)00230-8

[19] Zwartjes DG, Heida T, van Vugt JP, Geelen JA, Veltink PH. Ambulatory monitoring of activities and motor symptoms in Parkinson’s disease. IEEE Trans Biomed Eng. 2010;57: 2778–2786. 10.1109/TBME.2010.2049573 20460198

[20] Sim J, Wright CC. The kappa statistic in reliability studies: use, interpretation, and sample size requirements. Phys Ther. 2005;85: 257–268. 15733050

[21] Cohen J. Weighted kappa: nominal scale agreement with provision for scaled disagreement or partial credit. Psychol Bull. 1968;70: 213–220. 19673146 10.1037/h0026256

